# Impact of vaccination strategy adjustments on antibody levels against measles, rubella, mumps, and varicella in China: a single-center serological survey

**DOI:** 10.3389/fimmu.2025.1630442

**Published:** 2025-08-14

**Authors:** Zhixin Geng, Wen Wang, Yuanyuan Zhu, Yan Xu, Qiang Chen, Lei Zhang, Jing Yu, Xiang Sun, Zhiguo Wang

**Affiliations:** ^1^ Department of Laboratory Medicine, Jiangsu Province (Suqian) Hospital, Suqian, Jiangsu, China; ^2^ Department of Rheumatology and Immunology, Jiangsu Province (Suqian) Hospital, Suqian, Jiangsu, China; ^3^ Department of Expanded Program on Immunization, Jiangsu Provincial Center for Disease Control and Prevention, Nanjing, Jiangsu, China

**Keywords:** vaccination strategy, seroprevalence, measles, rubella, mumps, varicella

## Abstract

**Objective:**

This study evaluated trends in population immunity against measles, rubella, mumps, and varicella in Suqian City (2005–2024) using serological surveys (2019–2024) and incidence data, incorporating participants with known/unknown vaccination statuses to explore immunity dynamics amid evolving policies, and inform public health planning.

**Methods:**

Serum samples from 541 (2019) and 506 (2024) healthy participants were analyzed for virus-specific IgG antibodies using ELISA. Disease incidence data were obtained from China’s National Notifiable Disease Reporting System. Statistical analyses compared seroprevalence, geometric mean concentrations (GMCs), and incidence trends before and after policy adjustments.

**Results:**

Optimized two-dose strategies significantly improved seropositivity rates for rubella (65.1% to 70.8%), mumps (77.6% to 85.8%), and varicella (76.9% to 79.5%), with corresponding GMC increases. Incidence declines were notable: varicella (183.3/100,000 to 59.3/100,000), rubella (2.8/100,000 to 0.04/100,000), and mumps (24.4/100,000 to <5.0/100,000). However, measles seropositivity declined from 85.9% to 79.3% (p<0.05). GMC analysis showed increases for rubella (31.5 to 42.2 IU/ml), mumps (277.8 to 350.6 IU/ml), and varicella (295.9 to 309.8 IU/ml), but a decrease for measles (577.9 to 499.2 mIU/ml, p<0.001). Preschool children (2–5 years) exhibited the highest immunity levels in 2024.

**Conclusion:**

Population immunity against rubella, mumps, and varicella improved (2019–2024) with reduced incidence, while measles immunity declined, revealing vulnerabilities. Targeted strategies (e.g., catch-up campaigns for adolescents/adults, optimized infant vaccination) are needed to strengthen protection, considering interactions of policies, public health measures, and demographics.

## 1 Introduction

Vaccination remains the most effective tool for preventing infectious diseases and protecting public health (1). However, viral infections such as measles, rubella, mumps, and varicella continue to impose significant health burdens worldwide (2–5). Taking measles as an example, although global mortality rates have dramatically declined since 2000 through mass vaccination campaigns, persistent immunization gaps remain. According to the latest WHO report, global measles cases in 2023 reached an estimated 10.3 million, marking a 20% increase from 2022 and a notable resurgence compared to previous low incidence levels. Several countries experienced large-scale outbreaks, underscoring the risks associated with insufficient vaccine coverage (6, 7). Furthermore, the COVID-19 pandemic significantly disrupted routine immunization services (8, 9). Lockdown measures and healthcare resource reallocation during the pandemic led to substantial declines in vaccination rates across multiple nations (10). This reduction in immunization coverage directly exacerbated existing immunity gaps, triggering post-pandemic resurgences of vaccine-preventable diseases. Notably, upward trends in measles, rubella, mumps, and varicella cases have been observed not only in low-income countries but also in developed nations (11).

China has progressively optimized its vaccination strategies since the introduction of measles vaccine in 1965. The single-dose measles vaccination implemented in 1978 was replaced by a two- dose regimen (8 months and 7 years) in 1986, with the timing of the second dose moved earlier, to 18 months of age, in 2005. The national immunization program incorporated measles-rubella (MR) and measles-mumps-rubella (MMR) combination vaccines in 2007, and recommended two MMR doses at 8 and 18 months in 2020 (12, 13). Through five major immunization strategy adjustments from 1965 to 2020, China achieved remarkable progress in measles control, with incidence rates plummeting from 1432.4 per 100,000 in 1959 to 0.06 per 100,000 in 2020 (13). However, regional and temporal disparities in policy implementation persist. The 2019 measles outbreaks in certain areas revealed vulnerabilities in national immunity barriers. While varicella vaccine remained voluntary and self-funded with single-dose recommendation for decades, over half of Jiangsu Province’s cities adopted two-dose varicella vaccination in regional immunization programs starting 2020 (14), expanding province-wide by January 2023. Rubella, although largely controlled, remains a concern due to its ability to cause congenital rubella syndrome (CRS) in infants born to susceptible women. While rubella-containing vaccines have been integrated into China’s routine immunization schedule since the mid-2000s, immunity gaps persist among adolescents and adults born before its inclusion (15). Mumps is generally a self-limiting disease, but can result in complications such as orchitis, meningitis, and hearing loss. Outbreaks have been documented among school-age children and young adults, even among those with a history of one or two doses of mumps-containing vaccines, suggesting waning immunity or suboptimal vaccine effectiveness (16). Compared with countries employing MMRV (measles-mumps-rubella-varicella) quadrivalent vaccine strategies (17), China’s current single-vaccine approach may present limitations in enhancing compliance and achieving cross-protective immunity.

This study aimed to evaluate trends in population immunity levels against measles, rubella, mumps, and varicella in Suqian City, Jiangsu Province, China, across a 10-year period (2005–2024), using a dual cross-sectional serological survey (2019–2024) and disease incidence analysis. By incorporating participants with both known and unknown vaccination statuses, this study examines overall immunity dynamics in the context of evolving immunization policies, providing insights for future public health planning while recognizing the constraints of incomplete vaccination status data.

## 2 Materials and methods

### 2.1 Surveillance of measles, rubella, mumps, and varicella

In China, measles, rubella, and mumps are notifiable diseases monitored through the National Notifiable Disease Reporting System (NNDRS), a web-based platform that requires reporting of both clinically diagnosed and laboratory-confirmed cases. This surveillance system provided the data used to determine the incidence rates of measles, rubella, mumps, and varicella in Jiangsu Province analyzed in this study. Although varicella is not officially classified as a statutory notifiable disease, it is routinely surveilled and reported using the same protocols in practice in Jiangsu province. All reported cases are recorded by date of symptom onset and classified based on clinical diagnosis, laboratory confirmation, or epidemiological linkage, in accordance with national guidelines. Incidence rates are calculated per 100,000 population using demographic data from the National Bureau of Statistics. Since 1965, China has introduced a series of measles vaccination policy adjustments. These include the implementation of a single−dose schedule in 1978; a two−dose measles or measles–rubella schedule at 8 months and 7 years in 1986; rescheduling of the second measles−only dose to 18 months in 2005; and, in 2020, optimizing the program to a two−dose measles–mumps–rubella (MMR) schedule at 8 and 18 months, thereby introducing the more advantageous combined vaccine. For varicella, Jiangsu Province began piloting a two-dose schedule in select cities in 2020, with province-wide implementation by January 2023. These policy changes aim to improve population immunity and reduce the incidence of vaccine-preventable diseases.

### 2.2 Study participants

Serum samples were obtained from the Jiangsu Provincial Surveillance Program on Immunization Levels for Vaccine-Preventable Diseases in Healthy Populations. Suqian City served as the single-center study site. Residual serum samples collected from healthy individuals in 2019 (n = 541) and 2024 (n = 506) were analyzed, yielding a total of 1,047 samples. To ensure that seroprevalence reflected vaccine-induced immunity, individuals with recent vaccination (within six months) or a confirmed history of measles, rubella, mumps, or varicella infection were excluded. A stratified random sampling approach was used to enhance representativeness, with stratification based on age, sex, and geographic distribution across Suqian’s districts and counties. Age groups were defined as follows: 0–11 months, 12–23 months, 2–5 years, 6–18 years, 19–29 years, and ≥30 years. These corresponded to birth cohorts of approximately 2018-2019, 2017-2018, 2014-2017, 2001-2013, 1990-2000, and ≤1989 for the 2019 sample; and 2023-2024, 2022-2023, 2019-2022, 2006-2018, 1995-2005, and ≤1994 for the 2024 sample. Within each age group, samples were stratified by sex to ensure an approximately equal male-to-female ratio. Geographic stratification was conducted according to population distributions derived from the National Bureau of Statistics of China, with sample sizes allocated proportionally across administrative divisions. Random sampling was then performed within each stratum. Identical sampling procedures were used in both years to ensure comparability. For participants under 14 years of age, vaccination records were verified using official immunization documents or the Jiangsu Provincial Vaccination Integrated Service Management Information System. For individuals aged 14 years and older, vaccination history was primarily obtained through self-report, and participants with missing or unverified records were categorized as the “unknown” group. These participants were retained in the analysis to reflect real-world immunity levels, as their exclusion could introduce selection bias, especially in adult populations where vaccination records are often incomplete. Disease history was collected via participant recall.

### 2.3 Laboratory testing

Blood samples were collected from all participants. Serum was separated and stored at –20°C until analysis. IgG antibodies specific to measles, mumps, rubella, and varicella were measured using enzyme-linked immunosorbent assay (ELISA) kits (SERION ELISA classic, Institut Virion/Serion GmbH, Würzburg, Germany). All testing was performed at the central laboratory of the Affiliated Suqian First People’s Hospital of Nanjing Medical University. Quantitative analysis was conducted to determine seroprotection levels. Antibody concentrations were interpreted according to the manufacturer’s guidelines, which are based on internal validation and referenced literature. The cutoff values for seroprotection were as follows: Measles: ≥200 mIU/mL, Mumps: ≥100 IU/mL, Rubella: ≥20 IU/mL, Varicella: ≥50 mIU/mL.

### 2.4 Statistical analysis

Data were analyzed using Microsoft Excel and R software. Seroprevalence estimates were calculated with 95% confidence intervals (CIs) using the Clopper–Pearson exact binomial method. Geometric mean concentrations (GMCs) of measles- and rubella-specific IgG antibodies were summarized using medians and interquartile ranges [M (IQR)]. For comparisons of seroprevalence across ordered categories, the trend chi-square test was applied; for comparisons among unordered groups, the standard chi-square test was used. The primary dependent variables included binary IgG serostatus (positive/negative) for each virus and GMCs of virus-specific IgG antibodies. Independent variables were age group, sex, vaccination history, and survey year. Because antibody concentrations are often skewed or contain outliers, non-parametric tests (Wilcoxon rank-sum and Kruskal–Wallis) were used to compare GMCs, ensuring robustness and validity without assuming normality. Overall comparisons of seroprevalence and GMCs between 2019 and 2024 were conducted. Subgroup comparisons by age, sex, or vaccination history were not performed due to limited sample sizes and the need to reduce multiple testing bias. However, subgroup results are presented descriptively for transparency. All statistical tests were two-sided, and p-values <0.05 were considered statistically significant.

## 3 Results

### 3.1 Reported cases and incidence rates of measles, rubella, mumps, and varicella

**Figure 1:**
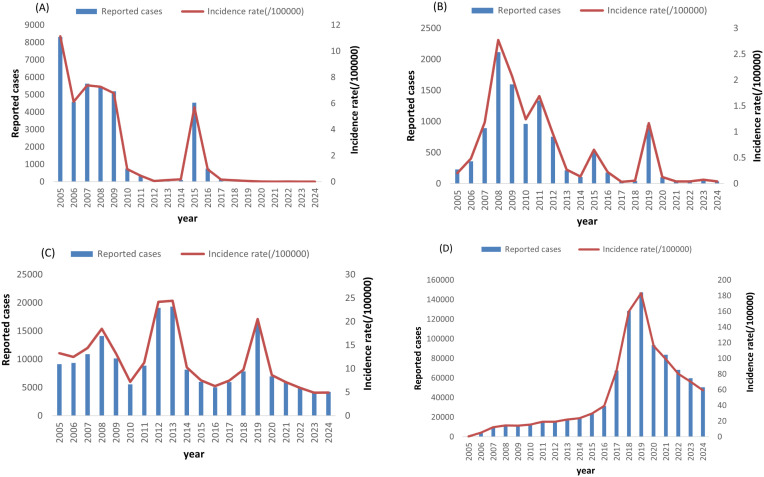
Reported cases and incidence of measles **(A)**, rubella **(B)**, mumps **(C)** and varicella **(D)** from 2005–2024 in Jiangsu Province, China.

As illustrated in **Figure 1**, Jiangsu Province exhibited significant declines in reported cases and incidence rates of varicella, measles, rubella, and mumps (2005–2024). Varicella incidence decreased from 183.3 per 100,000 in 2019 to 59.3 per 100,000 in 2024 (67.6% reduction). Measles incidence declined from 11.2 per 100,000 to 0.01 per 100,000 (99.9% reduction). Rubella incidence dropped from 2.8 to 0.04 per 100,000 (98.6% reduction), and mumps incidence fell from 24.4 to less than 5.0 per 100,000 (79.5% reduction).

### 3.2 Baseline characteristics of study participants 

A total of 541 samples were collected in 2019 and 506 in 2024. The sex distribution was balanced in both years, with males and females each accounting for about half of the participants. In both years, participants were distributed across all age groups. The proportion of children aged 6–18 years was higher in 2024 (30.4%) than in 2019 (22.2%), while the proportion of those aged 0–5 years was slightly lower in 2024. MMR vaccination history improved over time. In 2019, 10.5% of participants had not received any MMR dose, while in 2024, all participants had received at least one dose. The proportion of participants with two or more MMR doses increased from 36.4% in 2019 to 48.0% in 2024 (**Table 1**).

**Table 1:** Sample Distribution by Sex, Age, and Vaccination History in 2019 and 2024.

Characteristic	2019 (N=541)	2024 (N=506)	P-value
Sex			0.531
Male	273 (50.5%)	247 (48.8%)	
Female	268 (49.5%)	259 (51.2%)	
Age Group			0.032*
0-11 months	10 (1.8%)	5 (1.0%)	
12-23 months	62 (11.5%)	43 (8.5%)	
2-5 years	113 (20.9%)	88 (17.4%)	
6-18 years	120 (22.2%)	154 (30.4%)	
19-29 years	101 (18.7%)	82 (16.2%)	
≥30 years	135 (25.0%)	134 (26.5%)	
Vaccination History(MMR)			<0.001*
0 doses	57 (10.5%)	0 (0%)	
1 dose	37 (6.8%)	24 (4.7%)	
≥2 doses	197 (36.4%)	243 (48.0%)	
Unknown	250 (46.2%)	239 (47.2%)	

*a statistically significant difference between 2019 and 2024 (p < 0.05).

### 3.3 Seropositivity rates of measles, rubella, mumps, and varicella across different populations

**Figure 2:**
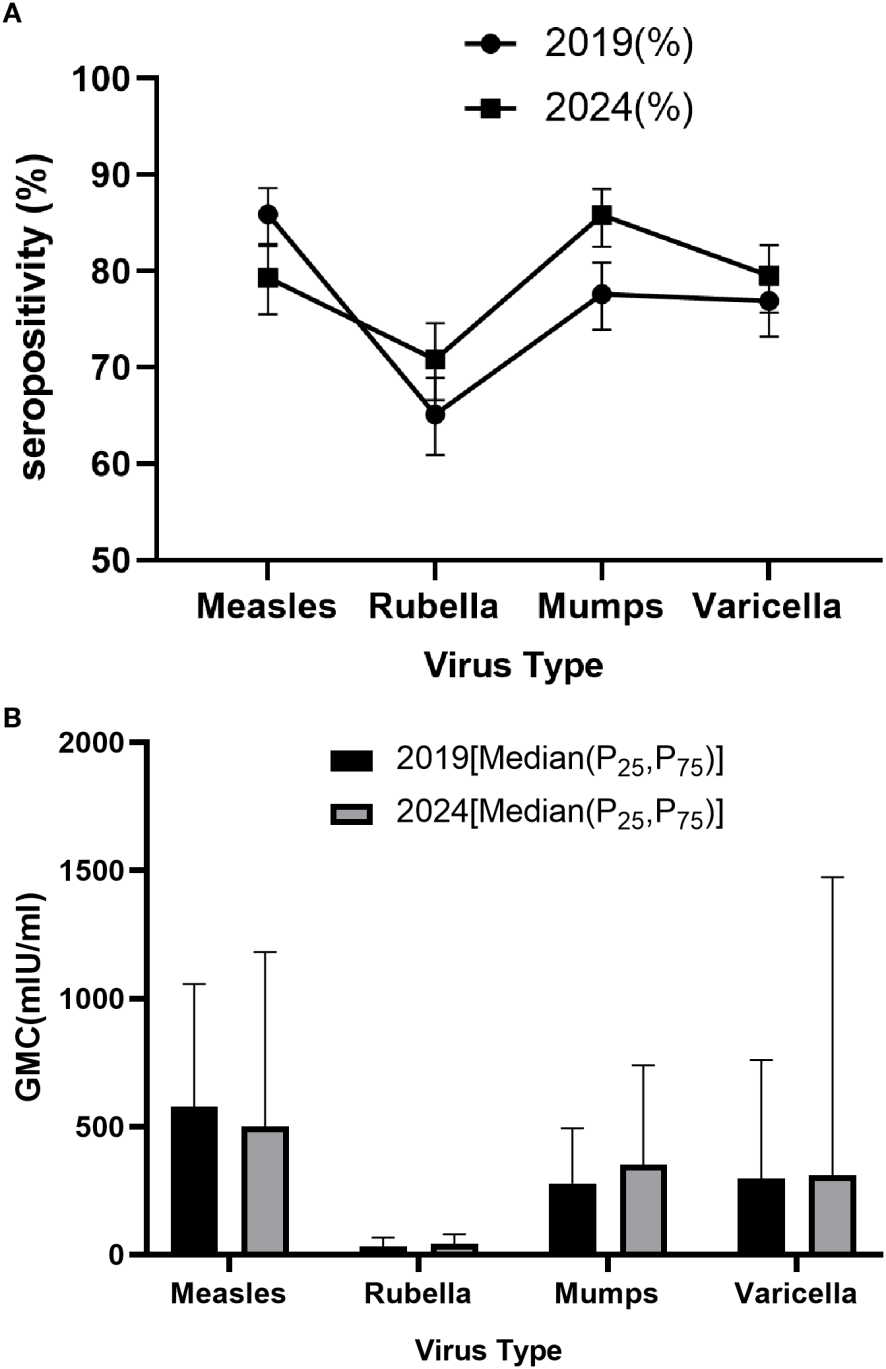
Seropositivity **(A)** and GMCs **(B)** of measles, rubella, mumps and varicella, 2019 and 2024.

In 2019, the seropositivity rates for measles, rubella, mumps, and varicella were 85.9%, 65.1%, 77.6%, and 76.9%, respectively. By 2024, significant increases were observed in rubella (70.8%) and mumps (85.8%) seropositivity (p<0.05), while varicella also rose to 79.5%(p>0.05), indicating improved population immunity against these three diseases. Notably, measles seropositivity declined from 85.9% in 2019 to 79.3% in 2024 (p<0.05), suggesting potential immunity gaps in measles protection. Gender analysis revealed no significant differences in seropositivity rates between males and females in either 2019 or 2024 (P>0.05), indicating minimal gender-based variations in immune responses to these four diseases. Age-stratified analysis showed significant differences among age groups (P<0.05), with children aged 2–5 years demonstrating higher seropositivity rates for all four diseases in 2024, while those under 2 years (particularly 0–11 months) exhibited lower overall seropositivity. Regarding vaccination history, following the immunization strategy adjustment, the measles seropositivity rate among recipients of two doses of measles-containing vaccine decreased from 94.4% in 2019 to 88.1% in 2024, while significant improvements were observed for rubella (84.8%), mumps (91.4%), and varicella (77.9%) seropositivity rates (**Tables 2A, B**, **Figure 2**).

**Table 2:** (A) Seroprevalence of measles, rubella, mumps in different groups in 2019 and 2024.

Characteristics	Measles					Rubella				
Survey year	2019 N positive, % (95%CI)		2024 N positive, % (95%CI)		P-value	2019 N positive, % (95%CI)		2024 N positive, % (95%CI)		P-value
Sex										
Male	227	83.1 (78.3-87.1)	188	76.1 (70.4-81.0)	0.049*	182	66.7 (60.9-72.0)	172	69.6 (63.6-75.0)	0.468
Female	238	88.8 (84.5-92.0)	213	82.2 (77.1-86.4)	0.035*	170	63.4 (57.5-68.9)	186	71.8 (66.0-76.9)	0.043*
P value	0.058		0.090			0.430		0.591		
Age										
0-11 months	1	10.0 (1.8-40.4)	2	40.0 (11.8-76.9)	0.494	2	20.0 (5.7-50.9)	2	40.0 (11.8-76.9)	0.494
12-23 months	50	80.7 (69.1-88.6)	16	37.2 (24.4-52.1)	<0.001*	44	70.9 (58.7-80.8)	12	27.9 (16.8-42.7)	<0.001*
2-5 years	99	87.6)80.3-92.5)	88	100.0	0.002*	81	71.7 (62.8-79.2)	84	95.5 (88.9-98.2)	<0.001*
6-18 years	108	90.0)83.3-94.2)	124	80.5 (73.6-86.0)	0.031*	75	62.5)53.6-70.6)	123	79.9 (72.8-85.4)	<0.001*
19-29 years	88	87.1 (79.2-92.3)	53	64.6 (53.8-74.1)	<0.001*	60	59.4 (49.7-68.5)	40	48.8 (38.3-59.4)	0.150
≥30	119	88.2 (81.6-92.6)	118	88.1 (81.5-92.5)	0.961	90	66.7 (58.4-74.1)	97	72.4 (64.3-79.3)	0.308
^a^P value	0.000		0.003			0.864		0.651		
Vaccination history										
0 dose	37	64.9 (51.9-76.0)	–	–	(N/A)	32	56.1 (43.2-68.2)	–	–	(N/A)
1 dose	35	94.6 (82.3-98.5)	0	–	<0.001*	27	72.9 (57.0-84.6)	2	8.3 (2.3-25.8)	<0.001*
≥ 2 doses	186	94.4 (90.3-96.9)	214	88.1 (83.4-91.6)	0.01*	135	68.5 (61.7-74.6)	206	84.8 (79.7-88.7)	<0.001*
unknown	207	82.8 (77.6-86.9)	187	78.2 (72.6-83.0)	0.186	158	63.2 (57.1-68.9)	150	62.8 (56.5-68.6)	0.950
^a^P value	0.263		0.946			0.547		0.001		
Total	465	85.9 (82.8-88.6)	401	79.3 (75.5-82.6)	<0.05*	352	65.1 (60.9-68.9)	358	70.8 (66.6-74.6)	<0.05*

^a^Linear-by-linear association in the chi-square test; *a statistically significant difference between 2019 and 2024 (p < 0.05).

**Table 2:** (B) Seroprevalence of mumps and Varicella in different groups in 2019 and 2024.

Characteristics	Mumps					Varicella				
Survey year	2019 N positive, % (95%CI)		2024 N positive, % (95%CI)		P-value	2019 N positive, % (95%CI)		2024 N positive, % (95%CI)		P-value
Sex										
Male	212	77.6 (72.4-82.2)	207	83.8 (78.7-87.9)	0.099	206	75.5 (70.0-80.2)	200	80.9 (75.6-85.4)	0.189
Female	208	77.6 (72.4-82.2)	227	87.6 (83.1-91.1)	0.011*	210	78.4 (73.1-82.9)	202	77.9 (72.6-82.6)	0.734
P value	0.990		0.217			0.424		0.408		
Age										
0-11 months	3	30.0 (10.8-60.3)	2	40.0 (11.8-76.9)	0.595	6	60.0 (31.3-83.1)	4	80.0 (37.6-96.4)	0.320
12-23 months	37	59.7 (47.3-70.9)	19	44.2 (30.4-58.9)	0.112	43	69.4 (57.0-79.4)	20	46.5 (32.5-61.1)	0.015*
2-5 years	85	75.2 (66.5-82.3)	80	90.9 (83.1-95.3)	0.001*	69	61.1 (51.9-69.6)	63	71.6 (61.4-79.9)	0.094
6-18 years	102	85.0 (77.5-90.3)	148	96.1 (91.7-98.2)	<0.001*	85	70.8 (62.2-78.2)	119	77.3 (70.0-83.2)	0.135
19-29 years	76	75.3 (66.0-82.6)	52	63.4 (52.6-73.0)	0.026*	86	85.2 (76.9-90.8)	72	87.8 (78.9-93.2)	0.601
≥30	117	86.7 (79.9-91.4)	127	94.8 (89.6-97.5)	0.017*	127	94.1 (88.7-96.9)	114	85.1 (78.1-90.1)	0.013*
^a^P value	0.000*		0.000*			0.000*		0.000*		
Vaccination history										
0 dose	35	61.4 (48.4-72.9)	–	–	(N/A))	23	51.1 (37.0-65.0)	6	60.0 (31.3-83.2)	0.637
1 dose	25	67.6 (51.5-80.4)	6	25.0 (12.0-44.9)	0.001*	134	69.8 (62.9-75.8)	48	56.5, (5.9-66.5)	0.047*
≥ 2 doses	164	83.3 (77.4-87.8)	222	91.4 (87.2-94.3)	0.006*	1	100.0	134	77.9 (71.1-83.5)	<0.001*
unknown	196	78.4 (72.9-83.1)	206	86.2 (81.2-89.9)	<0.001*	258	85.2 (80.7-88.7)	214	89.5 (85.0-92.8)	0.057
^a^P value	0.333		0.438			0.000		0.001		
Total	420	77.6 (73.9-80.9)	434	85.8 (82.5-88.5)	<0.05*	416	76.9 (73.2-80.3)	402	79.5 (75.7-82.7)	>0.05

^a^Linear-by-linear association in the chi-square test; *a statistically significant difference between 2019 and 2024 (p < 0.05).

### 3.4 GMC of antibodies against measles, rubella, mumps, and varicella in different populations

GMC analysis revealed that from 2019 to 2024, measles antibody levels decreased from 577.9 mIU/ml (IQR: 298.2-1056.3) to 499.2 mIU/ml (IQR: 220.6-1181.1) (p<0.001), while antibody levels increased for rubella (31.5 to 42.2 IU/ml), mumps (277.8 to 350.6 IU/ml), and varicella (295.9 to 309.8 IU/ml). Gender analysis showed no significant differences in GMC for measles, rubella, mumps, and varicella between males and females in both 2019 and 2024 (Wilcoxon rank-sum test, p>0.05). Age group analysis indicated significant differences in GMC across different age groups for all four diseases (Kruskal-Wallis H test, p<0.001). Specifically, in 2024, children aged 2–5 years achieved the highest GMC values: 1367.2 mIU/ml (IQR: 649.8-2869.5) for measles, 74.9 IU/ml (IQR: 41.4-122.6) for rubella, 570.6 IU/ml (IQR: 288.7-1039.3) for mumps, and 133.8 mIU/ml (IQR: 35.4-262.5) for varicella. Vaccination history analysis also showed significant differences in GMC among different vaccination dose groups for the four diseases (Kruskal-Wallis H test, p<0.001). The GMC trends aligned with seropositivity patterns, showing noticeable increases for all three diseases except measles, which demonstrated a slight decline (**Tables 3A, B**, **Figure 2**).

**Table 3:** (A) GMC of measles, rubella, mumps in different groups in 2019 and 2024.

Characteristics	Measles			Rubella		
Survey year	2019 Median (IQR:P_25_-P_75_) (mIU/ml, IU/ml)	2024 Median (IQR:P_25_-P_75_) (mIU/ml, IU/ml)	P-value	2019 Median (IQR:P_25_-P_75_) (mIU/ml, IU/ml)	2024 Median (IQR:P_25_-P_75_) (mIU/ml, IU/ml)	P-value
Sex						
Male	605.3 (286.5-1141.5)	552.9 (270.5-1338.0)	0.444	32.5 (13.2-66.0)	40.7 (14.5-72.1)	0.004*
Female	548.8 (308.7-996.1)	431.6 (206.5-1013.2)	0.045*	29.9 (13.2-70.2)	42.8 (17.9-84.4)	0.125
^a^P value	0.872	0.013		0.617	0.334	
Age						
0-11 months	49.9 (35.2-141.9)	192.4 (77.6-490.4)	0.129	4.3 (2.4-14.7)	16.3 (12.4-60.1)	0.040*
12-23 months	569.7 (159.2-1155.5)	97.3 (29.5-1585.9)	0.018*	46.1 (13.2-106.4)	7.2 (2.7-71.1)	0.001*
2-5 years	689.1 (347.9-1190.2)	1367.2(649.8-2869.5)	<0.001*	41.1 (14.1-88.3)	74.9 (41.4-122.6)	<0.001*
6-18 years	533.0 (313.7-1001.4)	392.7 (228.2-688.2)	0.002*	25.4 (15.4-49.6)	45.6 (23.1-71.7)	<0.001*
19-29 years	544.3 (298.9-857.0)	247.3 (162.8-408.9)	<0.001*	25.7 (8.8-58.3)	19.1 (6.9-46.9)	0.183
≥30	646.3 (314.5-1176.4)	749.3 (389.6-1359.5)	0.183	32.4 (13.6-64.5)	44.5 (18.1-91.4)	0.011*
^b^P value	0.000	0.000		0.000	0.000	
Vaccination history						
0 dose	286.9 (124.5-627.3)	–	–	24.4 (3.7-49.4)	–	–
1 dose	980.9 (440.0-1621.5)	32.7 (24.0-91.6)	0.265	58.3 (18.9-142.7)	2.9 (2.4-7.2)	0.132
≥ 2 doses	690.6 (373.8-1233.8)	644.7 (353.5-1491.4)	0.278	33.9 (16.2-78.5)	54.1 (28.1-91.2)	0.172
unknown	538.9 (257.5-992.6)	429.5 (215.1-934.3)	0.213	29.6 (10.3-57.9)	34.4 (12.6-62.7)	0.118
^b^P value	0.000	0.000		0.000	0.001	
Total	577.9 (298.2-1056.3)	499.2 (220.6-1181.1)	>0.05	31.5 (13.2-66.7)	42.2 (16.4-79.5)	<0.05*

^a^Wilcoxon rank-sum test;^b^Kruskal–Wallis H test; *a statistically significant difference between 2019 and 2024 (p < 0.05).

**Table 3:** (B) GMC of mumps and Varicella in different groups in 2019 and 2024.

Characteristics	Mumps			Varicella		
Survey year	2019 Median (IQR:P_25_-P_75_) (mIU/ml, IU/ml)	2024 Median (IQR:P_25_-P_75_) (mIU/ml, IU/ml)	P-value	2019 Median (IQR:P_25_-P_75_) (mIU/ml, IU/ml)	2024 Median (IQR:P_25_-P_75_) (mIU/ml, IU/ml)	P-value
Sex						
Male	285.9 (113.6-510.0)	310.9 (147.3-667.1)	<0.001*	397.2 (50.6-909.2)	438.9 (86.4-1471.6)	0.014*
Female	255.5 (115.4-474.6)	428.1 (180.9-834.0)	0.031*	271.4 (62.8-660.3)	280.8 (70.9-1527.3)	0.006*
^a^P value	0730	0.003		0.218	0.248	
Age						
0-11 months	58.5 (11.6-163.6)	74.9 (58.2-446.9)	0.254	91.5 (20.5-245.9)	600.4 (107.7-5069.4)	0.165
12-23 months	153.7 (32.9-402.8)	74.8 (24.3-406.9)	0.553	283.7 (31.8-711.3)	45.8 (24.7-234.8)	0.016*
2-5 years	304.6 (106.0-566.7)	570.6 (288.7-1039.3)	<0.001*	63.8 (25.9-479.1)	133.8 (35.4-262.5)	0.124
6-18 years	295.6 (141.2-537.9)	441.0 (200.3-938.9)	<0.001*	168.1 (41.9-714.9)	236.1 (68.3-1090.6)	0.087
19-29 years	236.3 (102.7-400.9)	171.9 (95.3-393.8)	0.424	464.6 (203.9-924.5)	363.5 (119.9-1647.3)	0.792
≥30	325.2 (152.4-507.5)	366.9 (221.5-619.8)	0.024*	562.2 (269.0-1010.7)	1580.5 (716.5-2932.5)	<0.001*
^b^P value	0.000	0.000		0.000	0.000	
Vaccination history						
0 dose	168.8 (39.5-402.5)	–	–	51.1 (14.9-91.9)	80.3 (36.2-1869.6)	0.097
1 dose	234.9 (46.2-406.9)	28.8 (21.9-107.0)	0.623	178.4 (33.5-618.5)	91.3 (24.7-304.4)	0.031*
≥ 2 doses	311.9 (142.9-577.3)	526.1 (246.5-1048.6)	0.325	–	203.9 (70.7-490.3)	–
unknown	267.3 (121.7-453.6)	296.5 (157.1-557.7)	0.025*	509.6 (198.2-913.9)	1023.8 (280.8-2213.9)	<0.001*
^b^P value	0.000	0.000		0.000	0.010	
Total	277.8 (115.2-493.4)	350.6 (164.7-739.2)	<0.05*	295.9 (54.9-759.9)	309.8 (80.7-1472.7)	<0.05*

^a^Wilcoxon rank-sum test; ^b^Kruskal–Wallis H test; *a statistically significant difference between 2019 and 2024 (p < 0.05).

## 4 Discussion

This study examined trends in population immunity against measles, rubella, mumps, and varicella in Suqian City, Jiangsu Province, China, between 2019 and 2024, using serological surveys and disease incidence data. Our findings reveal notable shifts in immunity levels: seropositivity rates and GMCs for rubella, mumps, and varicella increased, while measles immunity declined. These trends, accompanied by significant reductions in disease incidence, underscore the importance of maintaining robust population immunity to ensure collective protection.

The inclusion of participants with unknown vaccination status prevents us from directly attributing these immunity trends to specific vaccination strategies. Instead, the observed changes likely reflect a combination of factors, including natural exposure, overall vaccination coverage, and public health measures. For instance, varicella incidence dropped from 183.3 per 100,000 in 2019 to 59.3 per 100,000 in 2024, alongside improvements in seropositivity and GMCs. While this aligns with the implementation of a two-dose varicella vaccination strategy in Jiangsu Province, the lack of complete vaccination data limits definitive conclusions about its direct impact. Similarly, rubella and mumps seropositivity rates rose from 65.1% and 77.6% to 70.8% and 85.8%, respectively, with corresponding GMC increases and incidence reductions of 96.7% and 75.6%. These improvements coincide with global evidence on combined vaccines (18), but other factors—such as strengthened school-based prevention policies since 2019 and social distancing during the COVID-19 pandemic—may have contributed (19).

Measles presents a contrasting trend, with declines in seropositivity and GMCs. This may be linked to multiple factors: disruptions in routine immunization during the COVID-19 pandemic, waning immunity in older age groups (e.g., 19–29 years, where seropositivity fell from 87.1% to 64.6%), and reduced natural boosting due to extremely low disease prevalence (0.01/100,000 in 2024) (20). These findings suggest that unvaccinated or under-vaccinated populations remain vulnerable, highlighting the need for targeted catch-up campaigns to address age-specific immunity gaps (21).

Interestingly, no unvaccinated individuals were identified in the 2024 sample, and the proportion of one-dose MMR recipients decreased significantly since 2019. This could be explained by recruitment from routine immunization clinics, improvements in the Jiangsu Immunization Information System, post-pandemic MMR catch-up campaigns, and the younger age structure of the 2024 cohort, which likely benefited from higher two-dose coverage. Critically, by including participants with unknown vaccination status, our findings reflect real-world population immunity rather than isolated vaccine effects. Age-stratified analysis further revealed that preschoolers (2–5 years) exhibited the highest immunity levels, likely due to recent two-dose MMR completion by 18 months, reflecting policy effectiveness. While infants (0–11 months) showed low seropositivity and GMCs, indicating vulnerability before primary immunization. Conversely, adolescents and adults (born before 2005, not included in the EPI at birth) had lower rates of verified two-dose vaccination, particularly those aged 19–29 years, supporting the need for targeted catch-up strategies in these birth cohorts.

The study’s findings prompt consideration of alternative vaccination approaches, such as the MMRV quadrivalent vaccine used internationally (22, 23), which could reduce clinic visits and enhance compliance (24). However, challenges in China (25, 26), including limited domestic production capacity and regulatory hurdles, must be addressed to adopt such strategies. In the meantime, optimizing first-dose timing for infants and boosting immunity in older groups could strengthen population protection.

This study has some limitations. First, single-center design limits generalizability. Second, self-reported vaccination history risks recall bias. Third, subgroup comparisons were not statistically tested due to sample size constraints. Most importantly, by design, inclusion of participants with unknown vaccination status directs focus toward population immunity assessment rather than vaccine-specific effectiveness. Additionally, advanced methods like dynamic modeling and single-cell analyses could refine our understanding of immune response variability and inform precision immunization strategies (27–29).

## 5 Conclusion

Population immunity against rubella, mumps, and varicella improved from 2019 to 2024, accompanied by reduced incidence, while measles immunity declined, indicating potential vulnerabilities. These trends reflect complex interactions of immunization policies, public health measures, and demographic factors. Targeted strategies (e.g., catch-up campaigns for adolescents/adults, optimized infant vaccination timing, advanced MMRV vaccine evaluation) are needed to strengthen population protection.

## Data Availability

The raw data supporting the conclusions of this article will be made available by the authors, without undue reservation.
